# Zinc and thyroid cancer: A systematic review and meta-analysis protocol

**DOI:** 10.1371/journal.pone.0307617

**Published:** 2024-08-26

**Authors:** Aline Alves Soares, Yasmin Guerreiro Nagashima, Camila Xavier Alves, Kleyton Santos de Medeiros, Márcia Marília Gomes Dantas Lopes, José Brandão-Neto

**Affiliations:** 1 Postgraduating Program in Health Sciences, Federal University of Rio Grande do Norte, Natal, RN, Brazil; 2 Department of Nutrition, Liga Contra o Câncer, Natal, RN, Brazil; 3 Pesquisa e Inovação, Liga Contra o Câncer, Instituto de Ensino, Natal, RN, Brazil; 4 Department of Health Sciences, Federal University of Rio Grande do Norte, Natal, RN, Brazil; 5 Department of Nutrition.Federal University of Rio Grande do Norte, Natal, RN, Brazil; 6 Department of Internal Medicine, Federal University of Rio Grande do Norte, Natal, RN, Brazil; Dhaka University: University of Dhaka, BANGLADESH

## Abstract

**Introduction:**

The thyroid cancer has the ninth larger incidence of cancer in the world. Investigations related to the exposure to metals have become important due to the sensibility of the thyroid gland to them. Studies reveal that carcinogenic progressions are associated to the deficiency of the essential trace elements. In this context, the zinc is highlighted, essential for the metabolism of the thyroidal hormone and has a potential relation with the pathogenesis of the thyroid cancer. The objective of this systematic review and meta-analysis is to evaluate the low serum zinc as a risk factor for thyroid cancer in adults.

**Methods and analysis:**

PubMed/MEDLINE, Scopus, Embase and LILACS databases will be searched for observational studies investigating the low serum zinc as a risk factor for thyroid cancer in adults. No language or publication period restrictions will be imposed. The primary outcome will be that the low serum zinc is a risk factor for thyroid cancer. Three independent reviewers will select the studies and extract data from the original publications. The risk-of-bias will be assessed by using the Newcastle-Ottawa Quality Assessment Scale (NOS). Data synthesis will be performed using the R software (V.4.3.1) and to assess heterogeneity, we will compute the I2 statistic and the results will be based on either random-effects or fixed-effects models, depending on the heterogeneity. The Grading of Recommendations, Development, and Evaluation (GRADE) system will be used to evaluate the reliability and quality of evidence.

**Prospero registration number:**

International Prospective Register of Systematic Reviews (PROSPERO) CRD42023463747.

## Introduction

The thyroid cancer (TC) has the ninth larger incidence of cancer in the whole world [1, 2]. And if the recent tendencies are maintained, it can become the fourth most common cancer until 2030 in the United States [3].

There is a number of reasons responsible for this high incidence, as the enhancement of access to diagnostic procedures more intensive and sensitive. Nevertheless, it has been suggested that diagnostic technologies may not totally explain the growth in TC frequency, arguing that the environmental factors, lifestyle and comorbidities may contribute with this phenomenon [4–6]. The previous irradiation in the head/neck, history of benign thyroid nodules, goiter and family history of proliferative thyroid disease are risk factors established for TC [7, 8].

In addition, investigations related to exposure to metals have been becoming more important due to the sensibility of the thyroid gland to them. Studies reveal that carcinogenic progressions are associated to the excess of toxic metals (such as nickel, lead, cadmium), whereas the majority of the essential elements (selenium, zinc, magnesium) shows deficiency. This imbalance is capable of affecting the thyroid homeostasis because many of these trace elements are part of the metabolism of the thyroidal hormones, being an important risk factor in the development of TC [9, 10].

In this context, considering the health of the thyroid gland, among the essential trace elements, zinc (Zn) is highlighted, defined as a regulator metal in a number of aspects concerning the cellular function and metabolism. With Zn deficiency, multiple nonspecific general changes in metabolism and function occur, including reductions in growth, as well as the impairment of reproductive function and neurobehavioral development [11]. In addition, Zn is essential for the metabolism of the thyroidal hormone and has a potential relation with the pathogenesis of the TC [12]. Studies reveal that the Zn serum concentration is significantly reduced in many malignant tumors [13], including the TC. Specifically in the papillary thyroid carcinoma (PTC) and medullary thyroid carcinoma (MTC), the levels of serum Zn are lower than the ones found in healthy individuals [13, 14].

However, the results of studies concerning the Zn deficiency and TC are still inconsistent [13, 15, 16], showing that little is known about the role of Zn and the risk of progression of TC [9], preventing definitive recommendations.

In addition to the growing number of patients with TC^1^ and the inconclusive results of studies on Zn deficiency and TC risk [13, 15, 16], a study exploring the serum status of this trace element with greater depth is useful, as it is considered a vital component for the proper functioning of thyroid hormone metabolism and its deficiency can have a detrimental effect on thyroid activity [17].

Research with this objective may help understand the possible biological mechanisms involved in the deficiency of Zn and the thyroid carcinogenesis, helping the diagnosis and handling of patients with the worst prognoses. With that said, the objective of this systematic review and meta-analysis is to evaluate the low serum Zn as a risk factor for TC in adults.

## Materials and methods

The systematic review and meta-analysis will be conducted following the Meta-analysis of Observational Studies in Epidemiology (MOOSE) guidelines [18] and reported following the Preferred Reporting Items for Systematic Reviews and Meta-Analyses (PRISMA) statement [19, 20]. This protocol is listed in the International Prospective Registry of Systematic Reviews (PROSPERO) (CRD42023463747).

### Inclusion criteria

This systematic review and meta-analysis will include the following studies: observational studies (cohort, case-control, transversal) that evaluated the serum Zn a risk factor for TC; studies involving patients (age>18); with an apparently healthy population (in the controls for the case-control studies); studies without time restriction and studies published in any language.

### Exclusion criteria

The studies will be excluded if they are case reports, meeting abstracts, review papers and commentaries. Children and adolescents under 18 years of age will be excluded.

### The PECOT strategy

- Population: Adults (>18 years old)
- Exposure: Low serum zinc (12–16 μM Zn, equal to 0.785–1.046 mg/L) [21]
- Comparation: Adequate and/or elevated serum zinc
- Outcome: Thyroid Cancer
- Type of studies: Observational studies (cohort, case-control, transversal).

### Search strategy

The following databases will be used: PubMed/MEDLINE, Scopus, Embase and LILACS. No language or publication period restrictions will be imposed.

The Medical Subject Headings (MeSH) terms will be: ((Zinc) AND (Thyroid Neoplasm OR Neoplasm, Thyroid OR Thyroid Carcinoma OR Carcinoma, Thyroid OR Cancer of Thyroid OR Thyroid Cancer OR Cancer, Thyroid OR Thyroid Adenoma OR Adenoma, Thyroid) AND (Observational Study OR Cohort Study OR Retrospective Study)) (Table 1). The librarian participated in the development of the search strategy. The search strategy is shown in the S1 File.

**Table 1:** Presents the search strategy for Pubmed/MEDLINE.

	Pubmed/MEDLINE search strategy
**Search items**	
1	Zinc
2	1/AND
3	Thyroid Neoplasm
4	Neoplasm, Thyroid
5	Thyroid Carcinoma
6	Carcinoma, Thyroid
7	Cancer of Thyroid
8	Thyroid Cancer
9	Cancer, Thyroid
10	Thyroid Adenoma
11	Adenoma, Thyroid
12	3–11/OR
13	1 AND 12

### Other sources

The reference lists of the retrieved papers may also be used to choose appropriate research. In other words, the reference lists of the articles that were retrieved may allow the computerized literature search to be expanded. Identical strategies will be applied to other databases S1 File.

### Selection of studies

**Fig 1:**
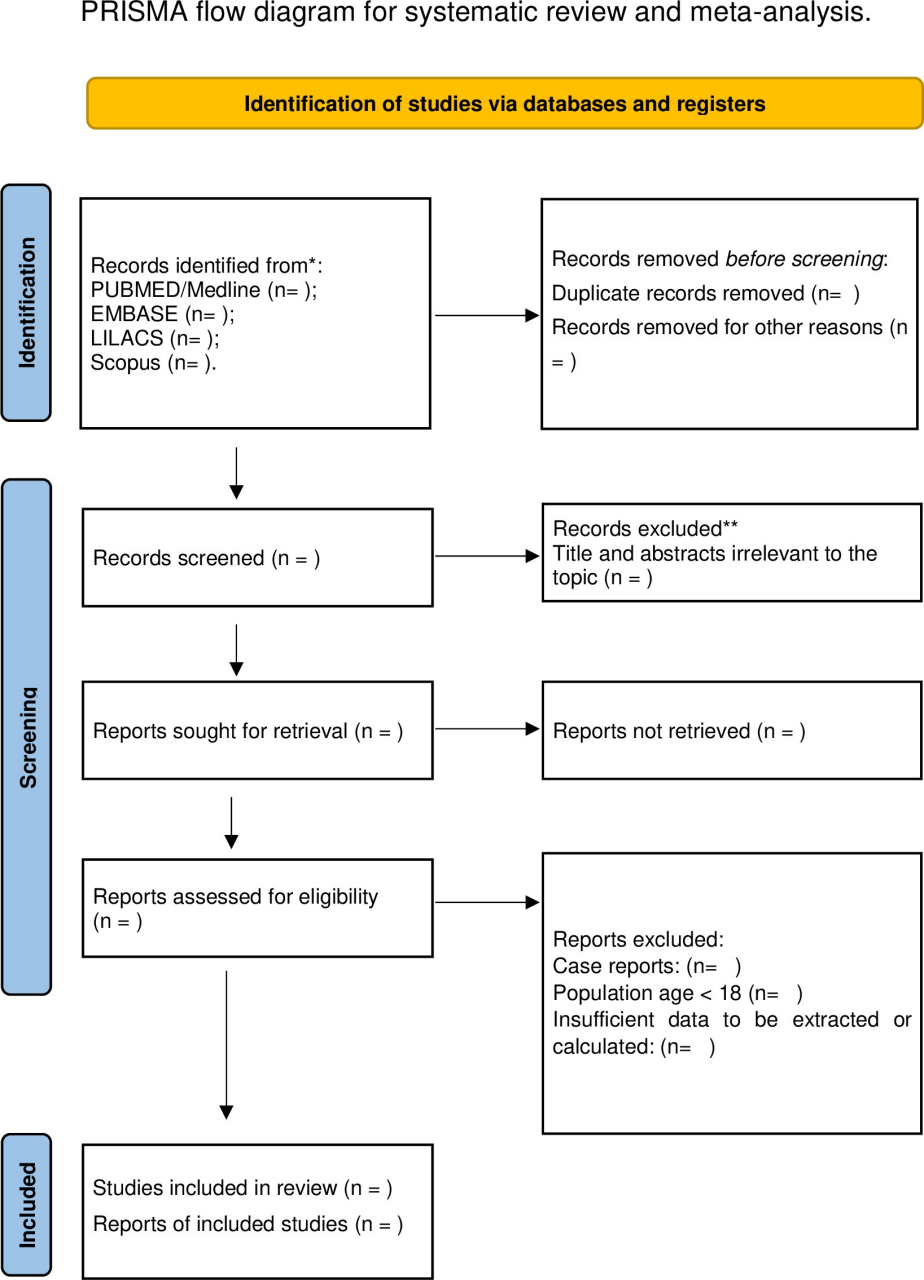
PRISMA flow diagram.

With Rayyan (https://www.rayyan.ai), two authors, AAS and YGN, will independently filter the search results based on titles and abstracts. Reviews and duplicate entries will be eliminated from the database. There will be an Excel table with the articles in it (Google Drive). To ascertain whether the research satisfy the inclusion criteria, the same authors will examine the entire text. Any differences will be resolved by CXA, the third reviewer. A PRISMA flow diagram will be used to summarize the chosen studies Fig 1.

### Data extraction and management

In accordance with the Cochrane tool, a standardized data extraction form will be created and evaluated. Two reviewers (AAS and YGN) will extract data separately from each included study and any inconsistencies will be discussed and addressed with a third reviewer (CXA). The data extracted will include information as the name of the first author; year of publication; country; sample size; gender and age of participants; number of participants in the case group (if case-control study); number of participants in the control group (if case-control study); kind of study; follow-up period; eligibility criteria; serum zinc levels; zinc measurement methods; quality control procedure of the serum Zn measurement; quantitative method of variable analysis. Likewise, we will extract the odds ratio (OR) and the 95% confidence interval (CI) for TC risk.

### Addressing missing data

Reviewers (AAS and YGN) will contact the authors or co-authors of the article if there are studies with missing, suppressed, or incomplete data. Communication will be via email. Additionally, supplementary documents related to the studies will be reviewed. If it is not feasible to obtain the necessary information, these studies will be addressed in the discussion section and excluded from the analysis.

### Risk of bias assessment

The bias risks of the included researches will evaluated independently by two investigators (AAS and YGN). The Newcastle-Ottawa Quality Assessment Scale (NOS) [22] will be utilized to evaluate the methodological quality of the studies. This evaluation tool comprises eight criteria that are grouped into three overarching perspectives: choosing the study groups, group comparability, and exposures or outcomes of interest. All things on the scale are given one point, or one star, with the exception of the item "Comparability", which has a score between zero and two stars. A study that is considered high quality will receive a rating of at least six stars; a study that is considered moderate quality will receive four or five stars; and a study that is considered low quality will receive less than four stars [22].

### Assessment of heterogeneity

A standard χ 2 test will assess the heterogeneity between the study outcomes at a significance threshold of p<0.1. We intended to compute the I2 statistic, a quantitative indicator of study inconsistency, to evaluate heterogeneity. Heterogeneity will only be assessed if a meta-analysis is warranted [23].

The I2 statistics <25% represented low heterogeneity, 25%-50%, moderate heterogeneity and >50%, high heterogeneity. In cases where there was substantial heterogeneity in the included studies (I2>50%), the random-effect model will be used, and when low heterogeneity exists in included studies the fixed-effects model will be used.

### Analysis

The R Software V.4.3.1 will be used to enter the data. The user can enter protocols, finish reviews, add text, research features, comparison tables, and study data, as well as carry out meta-analyses, with this software. The OR and 95% CI for each research will be extracted or computed for dichotomous data. The studies will be combined using the random-effects model in the event of heterogeneity (I2>50%), and the DerSimonian-Laird method will be used to get the OR and 95% CI. The robustness of the findings in relation to study quality and sample size will be investigated using sensitivity analysis. Only in the event that a meta-analysis is successful will this be feasible. In a summary table, the sensitivity analysis will be shown.

Considering the subgroup analyses, the assessment of serum Zn as a TC risk may be handled differently in the result analysis. The decision to perform subgroup analysis will take into account the heterogeneity and quantity of available studies. If a meta-analysis includes at least ten papers, we will attempt to perform subgroup analyses to account for any found heterogeneity among studies in order to provide for statistical power in these types of investigations. The nation, research type, age, gender, TC type, and Zn measuring techniques are the factors that will be taken into account.

If it is not possible to do a meta-analysis for all or part of the included studies, other research features and results will be narratively presented.

### Grading quality of evidence

The Grading of Recommendations Assessment, Development and Evaluation (GRADE) [24] method or a comparable approach that is properly stated and documented will be used to assess the degree of certainty in the evidence. The quality of evidence will be defined as “high”, “moderate”, “low,” and “very low” [24].

### Ethics and dissemination

Since this review will rely on publicly available scientific literature, ethical approval is not necessary. The results of this systematic review and meta-analysis will be published in a peer-reviewed publication and if sufficient new evidence becomes available to warrant a revision in the review’s conclusions, updates will be carried out. Any modifications to the protocol made while the review was being conducted will be noted in the manuscript.

## Discussion

Considering that the metal ions assemble in the thyroid and some play an important part in the function and homeostatic mechanisms of the thyroid gland, Zhou *et al*. [12] explain that alterations in some serums may be related to the pathogenesis of the TC.

Zn is a crucial trace element in the link of triiodothyronine (T3) with the nuclear receptor and is involved in the conversion of the thyrotropin-releasing hormone (TRH) to produce TRH via proteolytic conversion by a carboxypeptidase enzyme. The most important way towards the metabolism of thyroxine (T4) is through monodeiodination to produce the active thyroidal hormone, T3. This reaction is catalyzed by deiodinases type I and II (DI and DII) that need Zn as cofactor [25]. Therefore, the decrease of the Zn serum level may have a harmful effect over the thyroid activity that may be involved in the carcinogenic activity [17].

In this case, to help understand the biological mechanisms involved in the thyroidal carcinogenesis, this study was based in the evaluation of Zn serums in patients with TC.

Findings in Stojsavljević A. *et al*. [15] studies have indicated that the Zn (1613 ng/g) concentration average was significantly reduced (p<0.05) in blood samples of patients with TC when compared to the ones of the control group (5147 ng/g), result that may have an important role from the clinical point of view, for the purposes of diagnostics and traces. Analyzing other studies, similar outcomes support hypothesis that low Zn serums are associated to TC [16, 17].

The results of H. Al-Sayer *et al*. [16] and of Baltaci *et al*. [13] have discovered that the content of pre-operative Zn serum in patients with TC was significantly reduced when compared to a healthy one and that the surgical excision of the malignant thyroidal tissue has resulted in the restauration of the Zn content in regular amounts. Also, in the study by Baltaci *et al*. [13], measurements made immediately after the thyroid surgery have also shown lower levels of Zn serum in these patients (p<0.05). The surgical tissue though, indicated high average amounts of Zn. The fact that the same patients have presented lower zinc amounts in the serum samples indicates that this element is excessively withheld in the thyroidal tissue and can be related to the thyroid pathogenesis.

On the contrary, Rezaei M. *et al*. [26] couldn’t show any significant association between the Zn serum level and the risk of developing TC. The A. Emami *et al*. [14] study that sought to evaluate the status of micronutrients in Iranian patients with MTC before the thyroidectomy, has shown that the low Zn serum levels were not a risk factor for MTC.

Among types of TC, Bibi K and Shah MH [17] have compared the average Zn levels measured in the blood of various types of TC patients (anaplastic, follicular, medullary and papillary), identifying higher levels in anaplastic TC.

The results evidence the presence of altered Zn content in pathological blood samples in comparison to the control, indicating that the relation between Zn serum and TC is still controversial [13, 15, 16].

A systematic review and meta-analysis will help us to identify and synthesize the evidence of the association between Zn serum and TC. The results will also help us better understand the risk differences depending on gender, age, geographical location and types of TC. Also, a systematic review and meta-analysis about the matter will provide data about the methodology of different studies and the important points in published literature, which may help in the development of new experimental drawings, identifying the reasons of the discrepancies or contradictions between the results of the different investigations, encouraging the redrawing of the studies to improve the existing research methods.

The limitations of this review may involve the quality of primary studies, due to high methodological, clinical, and statistical heterogeneity among them. Especially, there is heterogeneity among the studies regarding Zn results and thyroid cancer risk, stemming from differences in social, demographic, and environmental factors, as well as variations in the types of TC among participants and characteristics of the measurement methods.

## Supporting information

S1 Checklist PRISMA-P 2015 checklist. (DOCX)

S1 File Search strategy in different databases. (DOCX)
