# Supplementary material for: Zinc and thyroid cancer: A systematic review and meta-analysis protocol
Source: PLoS One. 2024 Aug 26;19(8):e0307617. doi: 10.1371/journal.pone.0307617 (PMC11346916; doi:10.1371/journal.pone.0307617)
Supplement: S1 File — (DOCX) [file pone.0307617.s002.docx]

**Estrategy PUBMED (10/09/2023)**

(((Zinc[Mesh]) OR (Blood[Mesh]) OR (Serum[Mesh]) OR (Serum*) OR (Blood Serum*) OR (Plasma[Mesh]) OR (Plasma*) OR (Blood Plasm*) OR (Plasm*, Blood) OR (Fresh Frozen Plasm*) OR (Frozen Plasm*, Fresh) OR (Plasm*, Fresh Frozen) AND ((y_5[Filter]) AND (meta-analysis[Filter] OR observationalstudy[Filter] OR systematicreview[Filter]))) AND ((Thyroid Epithelial Cells[Mesh]) OR (Cell*, Thyroid Epithelial*) OR (Epithelial* Cell*, Thyroid) OR (Thyroid Epithelial* Cell*) OR (Thyrocyte*) OR (Thyroid Follicular* Cell*) OR (Cell*, Thyroid Follicular*) OR (Follicular* Cell*, Thyroid) AND ((y_5[Filter]) AND (meta-analysis[Filter] OR observationalstudy[Filter] OR systematicreview[Filter])))) AND ((Thyroid Neoplasms [Mesh]) OR (Thyroid Neoplasm) OR (Neoplasm*, Thyroid) OR (Carcinoma*, Thyroid) OR (Thyroid Carcinoma*) OR (Thyroid Cancer*) OR (Cancer*, Thyroid) OR (Cancer* of the Thyroid) OR (Adenoma*, Thyroid) OR (Thyroid Adenoma*) AND ((y_5[Filter]) AND (meta-analysis[Filter] OR observationalstudy[Filter] OR systematicreview[Filter])))

**Estrategy EMBASE via CAPES (24/06/2024)**

('thyroid tumor'/exp OR 'neoplasm of thyroid gland' OR 'neoplastic thyroid' OR 'neoplastic thyroid gland' OR 'thyroid gland neoplasia' OR 'thyroid gland neoplasm' OR 'thyroid gland tumor' OR 'thyroid gland tumour' OR 'thyroid neoplasia' OR 'thyroid neoplasm' OR 'thyroid neoplasms' OR 'thyroid tumorigenesis' OR 'thyroid tumour' OR 'thyroidal tumor' OR 'thyroidal tumour' OR 'tumor of thyroid' OR 'tumor of thyroid gland' OR 'tumor, thyroid gland' OR 'tumour of thyroid' OR 'tumour of thyroid gland' OR 'tumour, thyroid gland' OR 'thyroid tumor') AND ('thyroid follicular cell'/exp OR 'follicle cell, thyroid gland' OR 'follicular cell, thyroid' OR 'thyrocyte' OR 'thyrocytes' OR 'thyroid epithelial cell' OR 'thyroid epithelial cells' OR 'thyroid follicle cell' OR 'thyroid follicle cells' OR 'thyroid follicular cells' OR 'thyroid gland follicle cell' OR 'thyroid gland follicular cell' OR 'thyroid follicular cell') AND ('plasma'/exp OR 'blood plasma' OR 'human plasma' OR 'plasm' OR 'plasma preparation' OR 'plasma, blood' OR 'plasma' OR 'serum'/exp OR 'blood serum' OR 'concentrated serum' OR 'human serum' OR 'serum preparation' OR 'serum' OR 'zinc'/exp OR '64zn' OR 'zinc 64' OR 'zinc chelate' OR 'zinc content' OR 'zinc dust' OR 'zinc isotopes' OR 'zinc radioisotopes' OR 'zinc retention' OR 'zincum' OR 'zn' OR 'zn 64' OR 'zinc' OR 'blood'/exp OR 'blood drop' OR 'blood pool' OR 'blood product' OR 'human blood' OR 'human peripheral blood' OR 'peripheral blood' OR 'sanguis' OR 'tissue blood' OR 'whole blood' OR 'blood') AND [embase]/lim NOT ([embase]/lim AND [medline]/lim)

**Estrategy LILACS (24/06/2024)**

(mh:zinco OR (zinco) OR (zinc) OR (cinc) OR mh: d01.268.556.940* OR mh:d01.268.956.906* OR d01.552.544.940*) OR (mh:sangue OR (sangue) OR (blood) OR (sangre) OR (componentes do sangue) OR (hemocomponentes) OR mh:a12.207.152* OR mh:a15.145*) OR (mh:soro OR (soro) OR (serum) OR (suero) OR (sérico) OR mh:a12.207.152.846* OR mh:a15.145.846*) OR (mh:plasma OR (plasma) OR (plasma fresco congelado) OR (plasma liofilizado) OR (plasma sanguíneo) OR ma12.207.152.693* OR mh:a12.207.270.695* OR mh:a15.145.693*) AND (mh:"Células Epiteliais da Tireoide" OR (células epiteliais da tireoide) OR (thyroid epithelial cells) OR (células epiteliales tiroideas) OR (células foliculares da tireoide) OR (tireócito) OR (tireócitos) OR (células foliculares tiroideas) OR (tirocito) OR (tirocitos) OR mh:a11.436.911*) AND (mh:"Neoplasias da Glândula Tireoide" OR (neoplasias da glândula tireoide) OR (thyroid neoplasms) OR (neoplasias de la tiroides) OR (adenoma tireoidiano) OR (adenoma de tireoide) OR (carcinoma diferenciado de tireoide) OR (carcinoma tireoidiano) OR (carcinoma de tireoide) OR (câncer da glândula tireoide) OR (câncer da tireoide) OR (câncer de tireoide) OR (câncer de tireóide refratário a iodo radioativo) OR (neoplasia da tireoide) OR (neoplasias da tireoide) OR (neoplasias de tireoide) OR (tumor de tireoide) OR (tumores tireoidianos) OR (tumores de tireoide) OR (cáncer de la glándula tiroides) OR (cáncer de tiroides) OR (cáncer tiroideo) OR (neoplasias de la glándula tiroides) OR (tumores tiroideos) OR mh:c04.588.322.894* OR mh:c04.588.443.915* OR mh:c19.344.894* OR mh:c19.874.788*)

**Estrategy SCOPUS (25/06/2024)**

(("zinc") OR ("64Zn") OR ("zinc 64") OR ("zinc chelate") OR ("zinc content") OR ("zinc dust") OR ("zinc isotopes") OR ("zinc radioisotopes") OR ("zinc retention") OR ("zincum") OR ("Zn") OR ("Zn 64")) OR (("blood") OR ("blood drop") OR ("blood pool") OR ("blood product") OR ("human blood") OR ("human peripheral blood") OR ("peripheral blood") OR ("sanguis") OR ("tissue blood") OR ("whole blood")) OR (("serum") OR ("blood serum") OR ("concentrated serum") OR ("human serum") OR ("serum preparation")) OR (("plasma") OR ("blood plasma") OR ("human plasma") OR ("plasm") OR ("plasma preparation") OR ("plasma, blood")) AND (("thyroid follicular cell") OR ("follicle cell, thyroid gland") OR ("follicular cell, thyroid") OR ("thyrocyte") OR ("thyrocytes") OR ("thyroid epithelial cell") OR ("thyroid epithelial cells") OR ("thyroid follicle cell") OR ("thyroid follicle cells") OR ("thyroid follicular cells") OR ("thyroid gland follicle cell") OR ("thyroid gland follicular cell")) AND (("thyroid tumor") OR ("neoplasm of thyroid gland") OR ("neoplastic thyroid") OR ("neoplastic thyroid gland") OR ("thyroid gland neoplasia") OR ("thyroid gland neoplasm") OR ("thyroid gland tumor") OR ("thyroid gland tumour") OR ("thyroid neoplasia") OR ("thyroid neoplasm") OR ("thyroid neoplasms") OR ("thyroid tumorigenesis") OR ("thyroid tumour") OR ("thyroidal tumor") OR ("thyroidal tumour") OR ("tumor of thyroid") OR ("tumor of thyroid gland") OR ("tumor, thyroid gland") OR ("tumour of thyroid") OR ("tumour of thyroid gland") OR ("tumour, thyroid gland")) AND (("observational study":it) OR ("systematic review":it))
